# Multi-Stressed Nano and Micro-Silica/Silicone Rubber Composites with Improved Dielectric and High-Voltage Insulation Properties

**DOI:** 10.3390/polym13091400

**Published:** 2021-04-26

**Authors:** Faiza, Abraiz Khattak, Aqeel Ur Rehman, Asghar Ali, Azhar Mahmood, Kashif Imran, Abasin Ulasyar, Haris Sheh Zad, Nasim Ullah, Adam Khan

**Affiliations:** 1 School of Natural Sciences, National University of Sciences and Technology (NUST), Sector H-12, Islamabad 44000, Pakistan; faizaijaz710@gmail.com (F.); dr.azhar@sns.nust.edu.pk (A.M.); 2 Department of Electrical Power Engineering, U.S.-Pakistan Center for Advanced Studies in Energy, National University of Sciences and Technology (NUST), Sector H-12, Islamabad 44000, Pakistan; aqeelurrehman26@gmail.com (A.U.R.); kashifimran@uspcase.nust.edu.pk (K.I.); abasin@uspcase.nust.edu.pk (A.U.); 3 Department of Energy Systems Engineering, U.S.-Pakistan Center for Advanced Studies in Energy, National University of Sciences and Technology (NUST), Sector H-12, Islamabad 44000, Pakistan; asghar@uspcase.nust.edu.pk; 4 Department of Electrical Engineering, Riphah International University, Islamabad 46000, Pakistan; haris.shehzad@riphah.edu.pk; 5 Department of Electrical Engineering, College of Engineering, Taif University KSA, P.O. Box 11099, Taif 21944, Saudi Arabia; nasimullah@tu.edu.sa; 6 Department of Electronic Engineering, University of Engineering and Technology Peshawar, Abbottabad Campus, Abbottabad 22010, Pakistan; adamkhan@uetpeshawar.edu.pk

**Keywords:** silicone rubber, nanocomposite, dielectric properties, degradation, multi-stress

## Abstract

The scope of silicone rubber (SiR) is confined due to the deprivation of its dielectric propertiesupon exposure to various ambient stresses. The aim of this research is to develop silicone rubber-based composites by employing inorganic oxide fillers for improved dielectric and high voltage insulation properties for widening its scope in the field of electrical appliances. This study reports the preparation of different composites of silicone rubber with varying concentrations of micro and nano-silica fillers. The dielectric propertytrends of these as-prepared neat and impregnated samples were examined via an indigenously developed weathering chamber capable of applying multiple stresses of acid rain, heat, humidity, UVA radiation, and salt fog. Dielectric constant values were measured before and after applying stresses. Upon applying stresses, a periodic decline in dielectric constant was observed. Improved dielectric properties were obtained by adding micro and nano-silica as fillers. A nano silica-incorporated silicone rubber product exhibited good potential for dual applications as dielectric and high voltage insulation.

## 1. Introduction

Polymers are well known for their high insulation as well as stable physical and chemical properties. Nevertheless, polymer composites offer multiple improvements over simple polymers [1]. Polymer composites have an edge over conventional polymers due to their improved stability, greater lifespan, and enhanced physical, electrical, optical, and mechanical properties [2,3,4,5,6,7,8]. The development of charge-storing devices for higher storage capability needs to meet challenging criteria such as higher dielectric permittivity, low dielectric loss, and better mechanical strength [9]. Polymer composites provide low heat dissipation along with high permittivity. Metal filler-based polymer composites [10,11] have attained a huge concentration because of their light weight, low price, and easy processing. In the past, two types of approaches were adopted for polymeric dielectric composites [12]. The first method involved the addition of conductive fillers into the polymer network that formed a percolating system. This approach is based on the verdicts that very high permittivity can be achieved if filler volume is around a critical value. However, in this case, large dielectric loss was observed with low dielectric constant [13,14]. The second approach was evolved by adding ceramic insulating fillers. This method may give satisfactory results but, depending on size, higher loadings of fillers are required that compromise basic characteristics such as mechanical strength and flexibility [15,16,17]. Furthermore, in any approach, the uniform dispersion of fillers is of utmost importance to achieve the desirable properties [18]. Another drawback of polymer is that it degrades when it is exposed to environmental stresses, with temporary recovery and self-healing after the reduction in stresses [19,20]. It is evident from the literature that in some cases, composites showed improved resistance against ambient stresses and exhibited longer service life [21,22,23]. However, besides improvement in performance for insulation applications, polymers also need improved dielectric properties for enhanced charge storing capabilities.

Heat dissipation, which increases due to the aging effect, is a great challenge in charge-storing devices [24]. Therefore, there is a constant need of improvement in the dielectric properties of dielectrics [25]. Different fillers have been used in dielectric polymers for enhancement of their properties. For example, epoxy composites prepared by Zakya Rubab et al. along with the addition of sub-micron titania particles found an enhanced glass transition temperature as well as mechanical characteristics [26]. Zhou et al. focused on poly dimethyl siloxane (PDMS) incorporated with silicone carbide SiC and silicon nitride Si_3_N_4_ [27]. For thermal pad applications, Sim and co-workers examined the silicone rubber with inorganic fillers Al_2_O_3_ and ZnO that were conducive toward heat but electrically insulating [28].

Among different classes of fillers, inorganic oxides have been proved favorable for acquiring mandatory enlargement in nano/micro polymer dielectrics [29,30]. The literature shows that by the addition of inorganic fillers, an increased dielectric constant with low heat dissipation can be achieved. For example, QiangHe et al studied the thermo oxidative and swelling behaviors of cerium oxide/SiR composites and discovered the enhanced thermal resistance of SiR and improved re-interlinking ability of chains [31]. In a study on electrical treeing, SiR incorporated with Si_3_N_4_ and SiO_2_exhibited remarkable improvement [32]. Recently, a study on the dielectric characteristic and mechanical behaviors of chromium-based silicone rubber unveiled only minor improvement of 2 to 5 mass percent of filler [33]. A comparison of various studies revealed that among a broad array of inorganic fillers, silica is more cost-efficient and easily accessible, besides offering enhanced dielectric [34], mechanical [35,36], and electrical [37] characteristics. From the previous studies, it is obvious that numerous properties of silicon rubber enhanced by the addition of nano fillers [38,39], Ethylene propylene diene EPDM [40,41], epoxy [37,42], and Hydrogenated Nitrile Butadiene Rubber HNBR [43] can be achieved. Moreover, the degradation due to environmental stresses can be reduced.

To overcome the challenges presented by charge-storing devices, this work is dedicated to investigating the dielectric behaviors of silica-based silicone rubber micro and nanocomposites. The heat dissipation, dielectric constant, and conductivity are measured to check the stability and charge-storing ability of the material both before and after aging. This research will contribute in the area of charge-storing devices and modern insulation.

## 2. Materials and Methods

RTV-615A (base) and RTV-615B (curator) were obtained from General Electric Co, American Multinational corporation, Boston, MA, USA, Boston Lanxess Co, Cologne, Germany and Justus Kimiaraya, Jakarta, Indonesia. Nano-silica (AEROSIL^®^ 200) having a diameter of almost 12 nm was obtained from Degussa, USA and the specific surface area was 200 m^2^/g. Micro-silica having an average particle size of 5µmand surface area of ≥15 and ≤35 m^2^/g was procured from Wuhan New Reach chemicals China. Origin software was used to construct the graphs of properties.

### 2.1. Preparation of Samples

The samples were prepared according to weight (%) of the filler and polymer.For example, 5% nano-silica nanocomposite refers to 5 g of nano-silica filler and 95 g of silicon rubber. All the prepared samples were 80 mm in diameter with 3 mm of thickness. The ratio of base polymer to curator was 10:1. Before preparation, RTV-615A and B were kept in a vacuum for few hours and fillers were heated at 160 °C for 16 h. Thereafter, three major steps were followed to prepare the composites. In first step, dry fillers were added into RTV-615A at low mixing speed for wetting out the fillers. After wet mixing the fillers, speed was gradually increased to the maximum rpm until uniform dispersion was achieved. Then, the mixture was cooled for 2–3 min. In a second step, RTV-615B was added and agitated to achieve dispersion before being degassed in vacuum at 27 mm-Hg. In third step, the mixture was poured into the molds and kept at room temperature. The mixture was subsequently post cured for 4 h at 90 °C in the oven. The composition and codes of fabricated samples are tabulated as shown in Table 1.

### 2.2. Aging Setup

A chamber was developed according to the standards of the Electrical Power Research Institute (EPRI) and IEC-61109 with dimensions 1 m × 2 m × 1 m. The floor of the chamber was prepared from steel, while the walls were constructed with acrylic sheet [44,45]. The chamber was designed to provide multiple environmental stresses to the samples. Multiple stresses such as a test voltage of 2.5 kV, acid rain having 2.5 pH and salt fog of 5000 µS/cm were applied. For summer and winter cycles, temperature was kept at 47.2 and 35.3 °C, respectively. Acid rain was sprayed six times a day during summer and two times in winter. Exposure to UV-A radiations was for 10 h during summer cycles and for 8 h during winter cycles. Salt fog was only applied four times during winters. Each summer cycle was 11 days long, and each winter cycle was 17 days long. Total 28 weathering cycles were carried out continuously. The conditions employed during the experiment are summarized in Table 2 below [46].

### 2.3. Instruments and Conditions

#### 2.3.1. Scanning Electron Microscopy (SEM)

To ensure the proper dispersion of micro and nano fillers into the polymer matrix, a high-resolution scanning electron microscope, SEM Hitachi Ltd, Japan (Model no SU-1500), was employed. Material was coated with gold before subjecting to SEM analysis. In a polymeric composite material, especially in the case of rubber, reinforcement by filler greatly influences different properties. The filler–rubber interaction is explained by Fletcher and Gent [47], and the study was further enhanced by Payne [48]. In addition, the dispersion of filler by ionic liquids was discussed by Yasin et al. [49]. Basically, the particle sizes, amount of loading, and specific surface area are the main factors that determine the effective contact area between the rubber and filler. This interaction between rubber and filler could be physical or chemical. In the case of silica and silicon rubber, this interaction is chemical [50].

#### 2.3.2. Dielectric Properties

An Impedence analyser of Wayne Kerr company model no 6500B, West Sussex, UK was employed with a frequency range between 100 Hz and 5 MHz. A sample was prepared by cutting in a circular shape with 2 mm diameter and pressed between electrodes. The measurements were carried out under room temperature.

The dielectric constant is actually an ability of material to store charge at a specified frequency and temperaeture [51]. The dielectric constant was calculated as follows, where ε is the permitivity of the dielectric material and ε^o^ is the vaccum permittivity (8.85 × 10^−12^ F/m).

ε = ε/ε^0^ (1)

Despite a high dielectric constant (ε′), the material should maintain this property against applied multiple environmental stresses. Dissipation energy or dielectric loss(ε′′) is the [52] loss of electrical energy in the form of heating the medium, and it can be calculated as,

ε’’ = ε’(tanδ)(2)

where tanδ is the dissipation factor. During alteration, thepolarity charges displace from dielectric in one direction and then arranges it in the other direction. To overcome the opposition, they are exposed tothe produced heat; this phenomena is called dielectric loss.Since dielectric loss is directly related to the dielectric constant, an increase in frequency causes the value of dielectric lossto follow the same trend asobserved for the dielectric constant. The heat losses are high at lower frequencies because there is more resistance as compared to heat losses at higher frequencies [53]. The conductivity can be calculated as,

σ_ac_ = ωε^0^ε’(tanδ) (3)

To analyze the impact of silica and multiple weathering stresses on dielectric properties of neat SiR and its composites, dielectric constant (ε′), dielectric loss (ε′′), and AC conductivity (σ_ac_) are calculated before and after aging within 100 Hz to 5 MHz frequency range. 

## 3. Results and Discussion

### 3.1. Dispersion of Fillers and Other Additives

**Figure 1:**
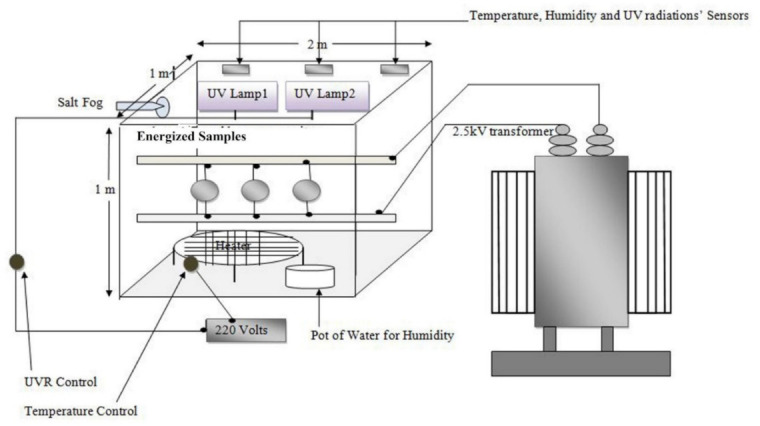
Schematic aging set-up model.

Silica is incoporated in silicon rubber as agglomerates; particularly, the migration of fillers plays an important role in vulcanized rubber: it detoriates the various properties of composites during their use Figure 1. To attain stability in desired properties of composites, the interfacial interactions between filler and rubber should be madiated crucially [54].

**Figure 2:**
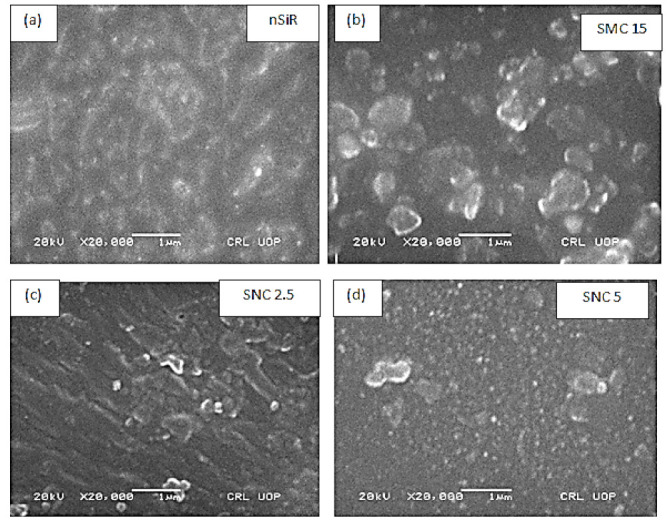
SEM images of (**a**) neat silicon rubber, (**b**) silica micro-composite (15% SiO_2_), (**c**) silica nanocomposite (2.5% SiO_2_), (**d**) silica nanocomposite (5% SiO_2_).

Therefore, good dispersion was achieved by using a high shear labortary mixer, and proper sonication was employed. SEM images of prepared samples are shown in Figure 2. It could be seen clearly that the proper dispersion of fillers was achieved and a better network with polymer was observed in the case of nano-silica as compared to the micro-silica. The interaction and dispersion of 5% nano-silica composite with silicon rubber was much better as compared to the 2.5% nanosilica and 15% micro-silica composite.

### 3.2. Dielectric Constant (ε′)

Dielectric constants were determined by the combination of ionic, electronic, and interfacial polarization. For polymeric materials, usually, a high dielectric constant is observed at low frequencies, butit decreases with an increase in frequency and eventually reaches a constant value. This trend can be explained by orientation of electric charge in one directionthat decreases with the increase in frequency. The higher dielectric constant of neatSiR and its other formulations is due to polar groups that align themselves with the applied electric field. In addition, infinite time is required for the charge carriers to align themselves. Therefore, as frequency increases, the dipoles fail to line up along their axes that are parallel to the applied AC field. As a consequence, the dielectric constant decreases with anincrease in frequency.

**Figure 3:**
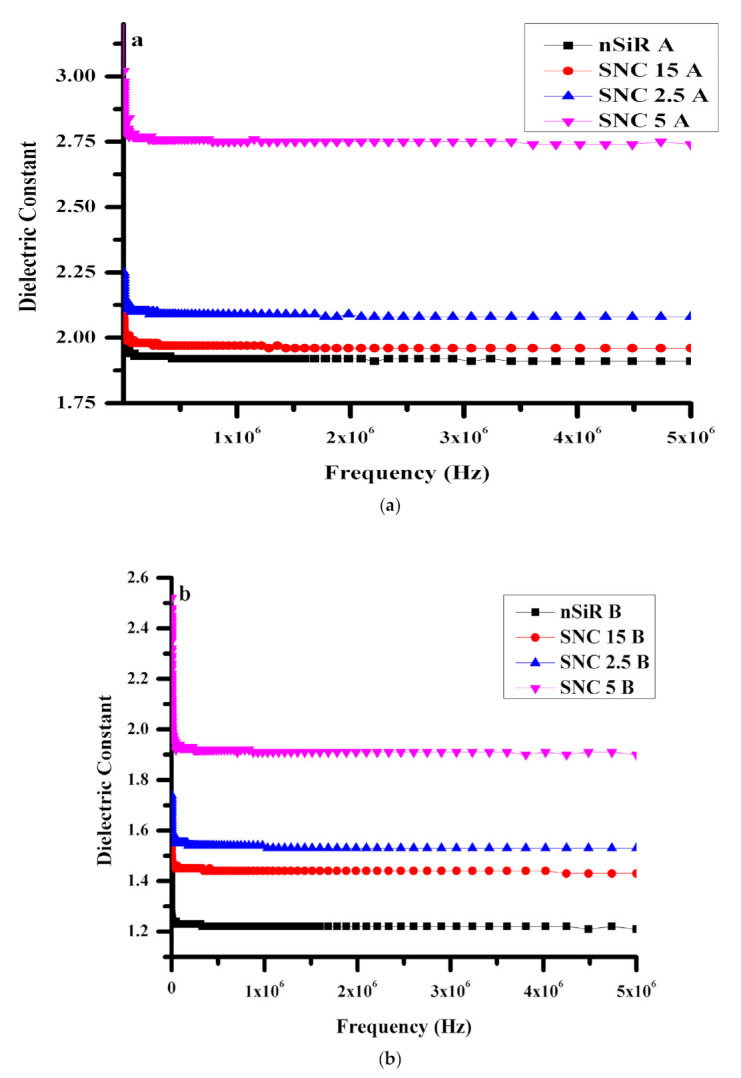
Dielectric constant (**a**) before aging, (**b**) after aging.

The dielectric constant (ε′) of neat silicone rubber and its composites with varying concenterations of micro and nano-silica fillers were analyzed before and after aging. An enhanced dielctric constant value due to micro and nano-silica fillerswas recorded, as depicted in Figure 3. The dielectric constant of unaged neat silicone rubber (nSiRA) was 2.20 at lower frequenciesbut became constant at 1.91 at higher frequencies. The micro-composite alsoexhibited the 2.20 value but reached a steady value of 1.96 at higher frequencies, which shows that the micro-composite has slightly more charge-storing ability, even at higher frequencies as compared to neatsilicone rubber. The micro-composites of SiR with micro-silica (SMC) showed a dielectric constantof 2.0 in the frequency range with a clear declining trend before 2.0 MHz but little decrease after 2.0 MHz. At a low frequency range, SiR with 2.5% nano-silica (SNC2.5) exhibited a little higher dielectric constant value of 2.25than both nSiR A and SMC15A, which decreased to 2.08 at a higher frequency range.Nevertheless, the 3.02 starting value for SNC5Awas not only superior than all other samples but also remain stable above 2.74 at higher frequencies. The above described trend of unaged samples is shown in Figure 3a.

Figure 3b shows dielectric constant trends of samples aged under multiple stresses. The values of dielectric constants for aged samples turned out to be lower than those of unaged samples. However, in the case of each sample concentration, the trend of a decreasing dielectric constant with increasing frequency was the same for both aged and unaged samples. This decrease was observed due to the degradation in grain boundaries. SNC5Bshowed the highest dielectric constant value of 1.98 among aged samples and remained above 1.90 for all frequencies. SNC2.5B exhibited a comparatively lower value of 1.74 and remained above 1.53. Over the entire explored frequency range, the dielectric constant of SMC15B remained lower than the corresponding values of SNC2.5 B and SNC5B but higher than that of nSiR B. By comparison, nSiR B showed the lowest dielectric constant of 1.37 at a lower frequency range that decreased to 1.21 at 5 MHz. This increase in values was seen as the filler concentration increased; this behavior was observed due to the fact that silica has more charge-storing ability than bare silicone rubber. Moving from micro to nanocomposites, results in fitting more filler particles per unit volume which in turn result in a greater dipole moment per unit volume and consequently higher dielectric constant values.

#### 3.2.1. Dielectric Loss (ε′′)

**Figure 4:**
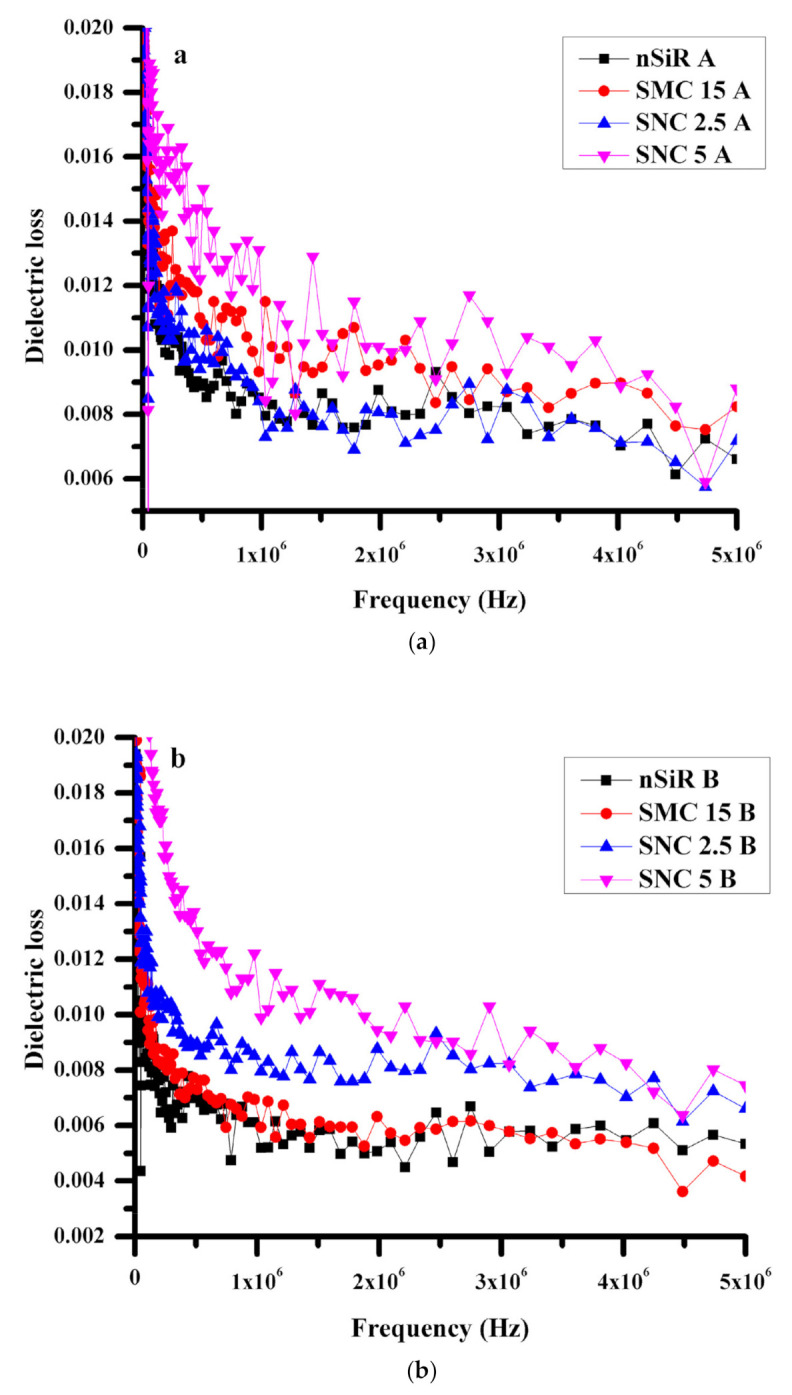
Dielectric loss (**a**) before aging, (**b**) after aging.

Generally, dissipation energy or dielectric loss enhanced with enhancement in dielectric constant, and the same trend was observed in all experiments performed for this research. As shown in Figure 4a, the dielectric loss of unaged samples was decreased with an increase in frequency in accordance with the above discussion. At lower frequencies between 100 and 200 Hz, the dielectric loss was less than 0.02, and a gradual decrease was recorded as the frequency increased. Moreover, at frequencies higher than 3 MHz, the heat loss ranged from 0.002 to 0.010. By comparison, for the lower frequency range between 100 and 200 Hz, aged samples have exhibited an average dielectric loss of 0.02, 0.018, 0.012, and 0.010 for SNC5B, SNC2.5B, SMC15B, and nSiR, respectively. Nevertheless, at frequencies above 1MHz, all the composite samples generally retained steady values within a small range, as shown in Figure 4b.

The unaged samples showed slightly higher heat dissipation than the aged sample because of the higher dielectric constant. Both SMC and nSiR have shown the same trend of higher dielectric loss in unaged samples. At very low frequencies, the dielectric loss for unaged neat silicone rubber was 0.02, but it decreased to 0.006and remained almost stable at frequencies above 200 Hz. For aged neat silicone rubber, the dielectric loss was 0.07, and after that, it became constant at 0.05. After aging, the difference was due to the decrease in the hopping of electrons as the material degraded. The same trend can be seen in case of SMC at lower frequencies. For both aged and unaged samples, the values of dielectric loss were the same at 0.02 at low frequency, but the values decreased to 0.008 and 0.004, respectively, as the frequency increased to 5 MHz. In contrast, SNC5 exhibited stability in dielectric loss value, as there was not much difference in the heat dissipation of both composites, even after applying aging conditions. At higher frequencies, loss factors were 0.006 and 0.008 for SNC2.5 and SNC5, respectively. It was discovered that by the addition of micro and nano fillers, a pronounced dielectric constant was achieved with low dissipation energy, and this effect was greater in the case of nanocomposites. SNC5 achieved the best dielectric constant with low dissipation energy and also showed stability even after applying environmental stresses. These results were obtained due to the greater intactness of backbone with filler in micro composite, and further enhancement was achieved due to the enhanced specific surface area when the filler size was reduced to the nanometer scale.

#### 3.2.2. Conductivity 

**Figure 5:**
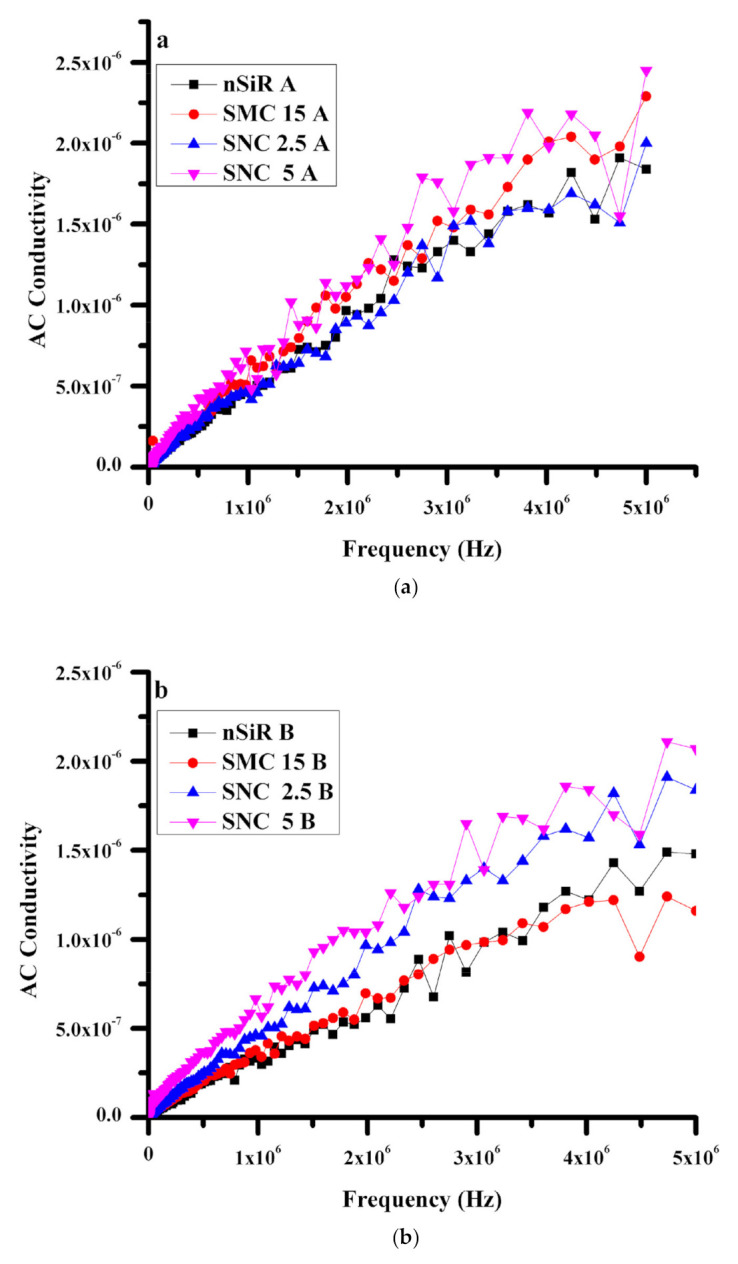
AC Conductivity (µS/cm) (**a**) before aging, (**b**) After aging.

Conductivity (σAC) increases with the increase in frequency due to disturbance in the placement of neighboring sites of cations. In general, the same increasing trend was observed in all samples. In unaged samples of SNC (5% and 2.5%), higher conductivity was observed than the conductivity values of neat silicone rubber and SMC, as illustrated in Figure 5a. A comparison of Figure 5a,b reveals that before aging, there was no significant difference in conductivity values, but after aging, the conductivity values of neat silicone rubber and the micro-composite were lower than those of the nanocomposites. Furthermore, the conductivity values of the nanocomposites agreed with the heat dissipation results.

For neat silicone rubber, at lower frequencies, the value of conductivity was 8.19 × 10^−10^ S/cm, which decreased to 4.16 × 10^−10^ S/cm after applying aging conditions. With the increase in frequency to 5 MHz, the value of conductivity was also increased to 1.84 × 10^−6^ S/cm for unaged samples and 1.48 × 10^−6^ S/cm for aged samples. The values were higher in the case of unaged samples due to more hopping of electrons, and the same trend was observed for all the samples. At a low frequency range, silicone rubber micro-composites showed 6.94 × 10^−10^ S/cm and 6.44 × 10^−10^ S/cm conductivities before and after aging, respectively. At a higher frequency range, conductivity became 2.29 × 10^−6^ S/cm for unaged samples and 1.16 × 10^−6^ S/cm for aged samples. Therefore, the micro-composite has demonstrated greater stability against stresses. Similarly, for SNC2.5 and SNC5, initially, the conductivity was 4.47 × 10^−10^ S/cm and 5.08 × 10^−10^ S/cm, respectively. However, at 5 MHz frequency, conductivity increased to 2 × 10^−6^ S/cm and 2.45 × 10^−6^ S/cm for SNC2.5 and SNC5,respectively. After applying aging conditions on SNC 2.5% and 5%, an initial conductivity of 8.19 × 10^−10^ S/cm and 1.36 × 10^−9^ S/cm was observed, respectively. Nevertheless, at 5 MHz, the conductivity rose to 1.84 × 10^−6^ S/cm and 2.07 × 10^−6^ S/cm for SNC 2.5% and 5%, respectively. It is critical to observe that the differences between conductivity values of aged and unaged samples of the nanocomposites are negligible as compared to the values for neat silicone rubber and the micro-composite.

## 4. Conclusions

SiR/silica nano (SNC) and micro (SMC) composites were prepared to examine the dielectric characteristics and permanence of dielectric parameters after aging under multiple environmental stresses. An environmental chamber was indigenously developed to apply multiple stresses such as salt fog, heat, acid rain, sunlight, and voltage. A scanning electron microscope was used to confirm the proper dispersion of filler into the polymer matrix. The dielectric parameters of aged and unaged samples were evaluated. SNC5A showed the highest dielectric constant value of 3.02, and even after aging, it retained the highest value of 1.98 amongst the aged samples. SNC2.5A showed a little higher value of 2.25 than SMC15A and nSiR. The dielectric constant was 2.20 for both the micro-composite and neat silicone rubber. However, the micro-composite showed a greater dielectric constant at higher frequencies. SNC2.5 and SNC5 both showed the same values for dielectric loss before and after applying aging conditions. Similarly, conductivity results also showed greater stability of nanocomposites after aging. Despite the high dielectric constant, SNC (5% SiO_2_) showed very low dissipation, similar to the other samples with low dielectric constant values, and it was almost negligible above 1MHz. The micro-composite has performed better than that of neat silicone rubber. Moreover, nanocomposites have depicted better dielectric behavior among all the samples, while SNC5 (5% SiO_2_) showed better dielectric results as compared to SNC2.5 (2.5% SiO_2_).

## Figures and Tables

**Table 1:** Concentrations of fabricated samples.

Sample	Code
Neat SiR	NS
SiR with 15% micro-silica	SMC15
SiR with 2.5% nano-silica	SNC2.5
SiR with 5% nano-silica	SNC5

**Table 2:** Applied conditions for multi-stress aging.

Applied Stress	Summer	Winter
Test voltage	2.5 kV	2.5 kV
Length of cycle (days)	11	17
Temperature (°C)	47.2	35.3
Ultraviolet-A (hours)	10	8
Acid rain (4.5 pH)	6 times	2 times
Salt fog (5000 µS/cm)	0 times	4 times

## Data Availability

Authors confirm the availability of all the supporting material and findings in the manuscript.
